# Typhoid Fever

**Published:** 1853-10

**Authors:** 


*Typhoid Fever.*—When meteorism, heat of skin, pain in the abdomen on pressure, and attacks of colic are the predominant symptoms in this disease, M. Sandras advises the application of ice to the abdomen. If nocturnal delirium, stupor, and congestion of the head occur, a bladder of ice should also cover the head. M. Sandras has long successfully employed this practice at the Beaujon hospital. The ice is mixed with linseed meal, which absorbs the water, and is frequently renewed. The tympanitis yields first; then the pain and fever gradually diminish. In the hemorrhages which occur during typhoid fever there is no better remedy than ice.—*Gazette des Hopitaux*, from *Ibid.*

